# Do Community Characteristics Explain Heat‐Related Illness in Seoul, Korea?

**DOI:** 10.1029/2025GH001580

**Published:** 2026-03-23

**Authors:** Minyeong Park, Jung Eun Kang

**Affiliations:** ^1^ Department of Urban Planning and Engineering Pusan National University Busan South Korea

**Keywords:** heat‐related illness, machine learning, community characteristics, climate risk assessment, apparent temperature, urban vulnerability

## Abstract

Heatwaves intensified by climate change have increasingly threatened public health, highlighting the need for proactive and spatially targeted interventions. This study aimed to provide scientific evidence for managing the risk of heat‐related illness (HRI) by integrating community‐level physical environments and sociodemographic characteristics and applying explainable artificial intelligence techniques. Based on the Hazard–Exposure–Vulnerability–Response framework presented in the IPCC Sixth Assessment Report, we evaluated 20 regression models using Seoul, South Korea, as a case study. Nonlinear models demonstrated superior predictive performance compared to linear models, and Shapley additive explanations analysis revealed that apparent temperature, urbanized area ratio, older‐adult population ratio, outdoor worker count, and accessibility to water areas were the most influential variables. Apparent temperature exhibited a distinct threshold with a sharp increase in HRI risk above 36°C, while less‐urbanized areas were associated with higher incidence rates. Communities with higher proportions of older adults and outdoor workers consistently demonstrated greater vulnerability, and the effect of accessibility to water areas was spatially limited within daily activity spaces. Given the weak linear associations between HRI incidence and all explanatory variables (*r* < 0.2), nonlinear dynamics and interactions play a critical role in understanding HRI risk. This study provides actionable insights for designing targeted heat‐health policies that consider diverse community characteristics.

## Plain Language Summary

This study identifies which communities in Seoul are more vulnerable to heat‐related illness (HRI) during extreme heat events. We found that higher apparent temperatures, lower levels of urbanization, and larger populations of older adults and outdoor workers were linked to higher rates of HRI. The results also suggest that nearby water bodies, such as rivers or streams, can help reduce heat risk, but only when they are easily accessible as part of people's daily activities. By examining complex relationships that are not well captured by simple linear analyses, this study helps explain how different community characteristics contribute to HRI risk. These findings can support decision‐makers in identifying high‐risk areas and implementing targeted actions—such as cooling shelters, shaded rest areas for outdoor workers—to better protect vulnerable populations during heatwaves.

## Key Points

- Community heat risk is shaped by thermal environment, exposure, vulnerability, and response at the neighborhood level
- Key drivers of heat‐related illness include apparent temperature, urbanization, older adults, outdoor workers, and water‐area accessibility
- Weak linear associations highlight nonlinear dynamics, supporting explainable machine‐learning approaches for heat‐related illness analysis

## 1 Introduction

Global average temperatures have been rising sharply because of climate change, making it increasingly urgent to develop strategies to address extreme weather events. According to the Intergovernmental Panel on Climate Change (IPCC) reports, the current global average temperature has increased by approximately 1°C compared to the pre‐industrial era, and heatwaves are projected to occur more frequently in various regions (Feng et al., 2022; IPCC, 2023; Mazdiyasni et al., 2017; Patel et al., 2022). This steady rise in global temperatures is intensifying both the frequency and severity of heatwaves, leading to a higher risk of heat‐related illness (HRI) during summer and posing significant public health threats.

HRI refers to a spectrum of conditions caused by prolonged exposure to extreme temperatures, including hyperthermia, heat edema, heat cramps, heat syncope, heat exhaustion, and heat stroke (Lipman et al., 2014). The human body temperature is strongly influenced by the environmental temperature. To maintain homeostasis, the body regulates its internal temperature at approximately 37°C by activating thermoregulatory mechanisms, such as sweating, increased heart rate, and faster breathing in response to heat. However, research has shown that for every 1°C increase in temperature beyond 29°C, the rates of respiratory‐related hospitalization and cardiovascular incidents increase significantly (Yang et al., 2021). Prolonged exposure to extreme heat can overwhelm thermoregulatory mechanisms, leading to such symptoms as excessive sweating, tachycardia, fatigue, dizziness, headaches, paresthesia, and muscle cramps (Beker et al., 2018).

For example, during a severe heatwave in Japan in 2018, approximately 95,000 people were hospitalized for HRI. In 2022, more than 60,000 deaths were linked to heatwaves in Western Europe (Ikeda & Kusaka, 2021; Zhao et al., 2021). The World Health Organization (WHO) has reported that 45% of heat‐related deaths occurred in Asia between 2000 and 2019, whereas 36% were recorded in Europe (WHO, 2024). These findings demonstrate the increased vulnerability of these regions to HRI.

HRI is a serious condition that can lead to coma or death in severe cases and is an important focus of research. Studies project that heat‐related mortality rates will rise further owing to climate change (Gould et al., 2024). Research also shows that vulnerable groups, such as older adults and those with cardiovascular or respiratory conditions, face higher rates of illness, hospital visits, and death (Arsad et al., 2022; Li et al., 2015). These findings highlight the need for further research on HRI and its impacts.

To prepare for the occurrence of HRI caused by heatwaves, it is essential to clearly understand the relationship between HRI and the factors influencing it. Previous studies have identified individual factors, such as age, occupation, and pre‐existing medical conditions, as key determinants of HRI (De Sario et al., 2023; Habibi et al., 2024; Hyatt et al., 2010). Several studies have analyzed the occurrence of HRI by considering urban climate, spatial structure, and socially vulnerable populations in cities (Ahn et al., 2024; Guirguis et al., 2018; Ovienmhada et al., 2024; Pyrina et al., 2025; Shindell et al., 2024). Given that such illnesses are sensitive to urban heat conditions and disproportionately affect socially vulnerable groups, it is crucial to examine both the physical environment and socioeconomic context of cities.

When HRI and its influencing factors are examined from a multidimensional perspective, physical‐environmental and socioeconomic variables may exhibit not only simple linear relationships but also complex non‐linear associations. For example, Yang et al. (2025) used gradient boosting and Shapley additive explanations (SHAP) to demonstrate that nighttime light intensity, floor area ratio, and the proportion of older adults—representing urban heat vulnerability—have non‐linear effects on heat‐related morbidity risk. Foroutan et al. (2025) developed a geospatial AutoML–XAI model to quantify the relative contributions of meteorological, land‐cover, and socioeconomic indicators to heat‐related emergency department visits in Texas. Additionally, Guo et al. (2025) applied a machine‐learning approach based on Local Climate Zones and revealed that high‐density, highly impervious development and socioeconomic vulnerability are key drivers of increased urban heat–health risk and spatial inequity. These studies collectively demonstrate that traditional regression methods alone are insufficient to explain the complex and non‐linear relationships between HRI and its determinants, highlighting the effectiveness of machine‐learning approaches, including explainable artificial intelligence, as a valuable alternative.

Building on this background, this study aims to identify the key factors associated with community‐level incidence of HRI by examining both linear and non‐linear relationships between environmental and socioeconomic determinants. To this end, an explainable machine‐learning approach was applied to enhance the interpretability of model results.

## 2 Risk in the Context of Climate Change

In the IPCC Fourth Assessment Report, assessments of climate change impacts primarily adopted a vulnerability‐based approach (Lee et al., 2016). In this framework, vulnerability was determined by three components—exposure, sensitivity, and adaptive capacity—and assessments focused on the socioeconomic conditions that influence how severely a given region may be affected by climate change (IPCC, 2007, p. 32). However, this approach has limitations, as it does not adequately account for the full range of climate change impacts.

Recognizing these shortcomings, the IPCC Fifth Assessment Report emphasized the need for a paradigm shift from vulnerability assessments to risk assessments (IPCC, 2014, p. 3). According to the IPCC, climate risk is determined by three key components: hazard, exposure, and vulnerability. Furthermore, the IPCC Sixth Assessment Report (AR6) highlighted the importance of considering response as a means to mitigate risk arising from the interaction of these components (IPCC, 2023). In this context, response refers to policy actions aimed at reducing climate risk and can be broadly categorized into mitigation—reducing greenhouse gas emissions and facilitating a transition to a low‐carbon society—and adaptation, which focuses on enhancing resilience and reducing vulnerability in social systems and ecosystems.

Following the introduction of these frameworks, numerous studies have applied either the risk framework or the earlier vulnerability framework to examine climate‐related impacts. The following section summarizes the variables used in previous studies for each risk component.

## 3 Heatwave Risk

In the context of heatwave risk and vulnerability assessments, numerous studies have used various indicators to evaluate the impacts of heatwaves across different regions. This study categorizes indicators used in previous research into four groups, based on the three risk determinants defined in the IPCC risk framework and an additional response component that addresses these determinants.

Hazard, which refers to natural disasters or meteorological phenomena resulting from climate change, is commonly represented by such indicators as the number of heatwave days, number of tropical nights, urban heat island intensity, average temperature, maximum temperature, heatwave frequency, relative humidity, apparent temperature, and discomfort index (Ahn et al., 2024; Kim, 2021; Tomlinson et al., 2011; Zhang et al., 2021). These indicators reflect the intensity and persistence of hazards, with higher values corresponding to higher risk levels. In this context, Ahn et al. (2024) emphasized the need for climate‐specific thermal indices, showing that the Wet‐Bulb Globe Temperature (WBGT) demonstrates higher predictive accuracy in arid climates, whereas the Heat Index performs better in humid climates.

Indicators of exposure are inventories of elements exposed in hazardous areas, reflecting the extent to which populations or facilities are present in such areas (IPCC, 2023). Examples of exposure indicators include population density, total population, and the urbanized area ratio. Grigorescu et al. (2021) classified potential exposure factors into socioeconomic exposure and environmental exposure to evaluate heat‐related vulnerability. For socioeconomic exposure, they selected such variables as population with permanent residency and agricultural land area, whereas for environmental exposure, they included impervious surface area and green space area. In Grigorescu et al. (2021), higher values for population, agricultural land area, and impervious surface area were associated with increased vulnerability, whereas larger green‐space areas were associated with reduced vulnerability. Su et al. (2024) and Ren et al. (2025) also used WorldPop Hub population data as exposure indicators, which include population density information disaggregated by sex and age groups. In addition, Zitouni et al. (2025) applied a risk assessment framework and included both population density and the Normalized Difference Building Index (NDBI) as exposure indicators, with NDBI representing built‐up areas and the degree of urbanization.

In the context of vulnerability, commonly used indicators include the population aged 65 years or older, children under 5 years, single‐person households, older adults living alone, the proportion of people with education below higher education, poverty levels, the proportion of outdoor workers, and the population with disabilities. These indicators reflect populations that are particularly vulnerable to heatwaves compared with the general population. These variables are generally interpreted as indicating increased vulnerability, with higher values corresponding to increased risk. Other studies have included such indicators as unemployment rate and the number of people with pre‐existing health conditions (Grigorescu et al., 2021; Lee et al., 2020; Tomlinson et al., 2011). In multi‐ethnic countries, indicators related to racial/ethnic minority populations have also been incorporated (Conlon et al., 2020).

In terms of response, commonly used variables include road area, canopy cover, park and green space area, accessibility to water bodies, accessibility to potable water, density of health centers, emergency rooms, fire stations, heat shelters, number of disaster‐related public officials, gross regional domestic product (GRDP), fiscal independence, number of health insurance subscribers, and number of healthcare workers. These indicators reflect the facilities or support systems available in a region that mitigate the risks associated with heatwaves.

Response indicators have been consistently applied in previous studies, with lower values corresponding to increased heatwave risk. Among these, parks and green‐space areas, as well as accessibility to water bodies, were the most frequently utilized indicators. Although the formats of these data, such as the normalized difference vegetation index, land cover classification, and zoning data, vary, they have been widely used to emphasize the role of green spaces and water bodies in mitigating high‐temperature environments and reducing heatwave risks (Conlon et al., 2020; Grigorescu et al., 2021; Zhang et al., 2021).

Examples include road area, density of health centers, emergency rooms, fire stations, number of health insurance subscribers, and healthcare workers, which have often been used in studies examining regional inequalities (Kim, 2021). These variables have been interpreted as indicators of healthcare capacity. Similarly, GRDP, fiscal independence, and the number of disaster‐related public officials have been analyzed as indicators of a region's financial response capacity, with higher values associated with reduced heatwave risks (Zhang et al., 2021).

## 4 Methods

### 4.1 Study Area and Spatial Unit

**Figure 1:**
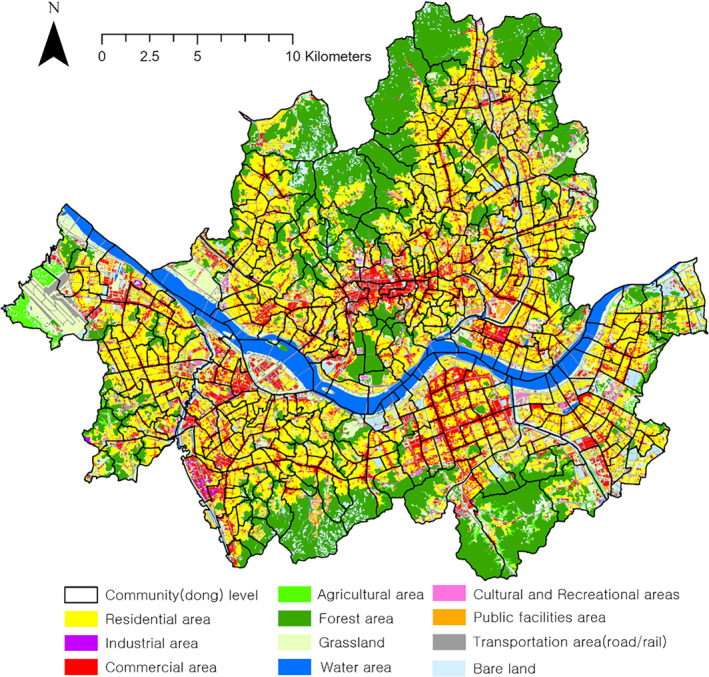
Study area.

This study focuses on Seoul (see Figure 1), South Korea, as the target area. Seoul, the most densely populated city in the country, faces significant climate‐related challenges, including the urban heat island effect. Furthermore, the city provides extensive public data, making it one of the most data‐rich urban regions for empirical research. These factors enhance the reliability of the analysis and increase the applicability of the findings to policymaking; accordingly, We selected Seoul as the study area.

The spatial unit of analysis is the community‐level unit known as “*dong*” (the smallest administrative division) in Seoul, which comprises 425 units. Because data collection occurs at the community level, this unit enables fine‐grained analyses that can be directly applied to policy decisions. The size of a *dong* varies widely, ranging from 0.2 to 12 km^2^, with populations between 1,000 and 50,000 residents.

Figure 1 illustrates the dong‐level administrative boundaries and land‐cover distribution in Seoul. The land‐cover data were obtained from the 2024 medium‐classification data set provided by the Environmental Geographic Information Service of Korea. The Han River runs across the central part of the city, with several smaller tributaries extending throughout the urban area. Forested areas are primarily located in the northern and southern parts of Seoul, while expansive cropland and grassland are observed in the western and eastern outskirts. Residential and commercial areas are widely distributed across the city, excluding the forested zones and river corridors. These spatial characteristics provide essential baseline information for understanding the spatial disparities in thermal environments and heat‐related health risks across Seoul. In particular, forested areas and river systems serve as important moderators of urban heat conditions.

### 4.2 Data

This study assembled a set of variables representing the characteristics of urban regions based on the Hazard–Exposure–Vulnerability–Response framework. The explanatory variables were categorized into 16 indicators across these four components.

*Hazard*: two indicators (maximum temperature, apparent temperature)
*Exposure*: two indicators (population density, urbanized area ratio)
*Vulnerability*: six indicators (older‐adult population ratio, infant and toddler population ratio, low‐income group ratio, older‐adult living alone ratio, disability population ratio, and outdoor worker count)
*Response*: six indicators (accessibility to parks, accessibility to medical facilities, accessibility to pharmacies, accessibility to green spaces, accessibility to water areas, accessibility to heat shelters)

The dependent variable was the incidence rate of HRI, which was calculated for each dong based on the number of HRI cases. Using the total number of cases as the dependent variable posed a risk of bias owing to the inherent correlation between population density and case counts, potentially skewing the analysis. By contrast, the incidence rate allowed for comparisons between regions regardless of population size, thereby enhancing the validity of the analysis. The data on HRI cases were obtained from the National Health Insurance Service (NHIS) of Korea. Cases were identified based on Korean Standard Classification of Diseases (8th revision) using code T67. This category encompasses a range of heat‐related conditions, including heat stroke, heat exhaustion, heat syncope, heat cramps, transient heat fatigue, and heat edema. This data set provides the number of HRI cases by dong and month. The incidence rate of HRI was calculated by dividing the number of cases by the corresponding population in each dong.

The data covered a 7‐year period from 2013 to 2019, and the analysis was restricted to the summer months (June–August). When data for a specific year were unavailable, values from the nearest available year were used as substitutes. Among the explanatory variables, maximum temperature, apparent temperature, population density, infant and toddler population ratio, and older‐adult population ratio vary by dong and month. The remaining explanatory variables vary by dong and year. The dependent variable—the incidence rate of HRI—also varies by dong and month. A detailed description of the data used in this study is provided in Table 1.

**Table 1:** Summary of Dependent and Explanatory Variables

	Risk assessment components	Detailed indicators (community characteristics)	Explanation	Sources
Explanatory variables	Hazard	Maximum temperature	Monthly maximum daily temperature at the administrative dong level	Korea Meteorological Administration Data Portal
		Apparent temperature	Monthly average apparent temperature calculated using daily maximum temperature and average humidity based on Stull's formula, at the administrative dong level	
	Exposure	Population density	Monthly population density per 10,000 m^2^ at the administrative dong level	Statistics Korea
		Urbanized area ratio	Annual ratio of urbanized areas (land cover major class code 100) at the administrative dong level	Environmental Geographic Information Service
	Vulnerability	Older‐adult population ratio	Monthly number of people aged 65 years or older per 10,000 population at the administrative dong level	Statistics Korea
		Infant and toddler population ratio	Monthly number of children aged 4 years or younger per 10,000 population at the administrative dong level	
		Low‐income group ratio	Annual number of National Basic Livelihood Security recipients per 10,000 population at the administrative dong level	Seoul Open Data Plaza
		Older‐adult living alone ratio	Annual number of older adults living alone per 10,000 population at the administrative dong	
		Disability population ratio	Annual number of individuals with disabilities per 10,000 population at the administrative dong level	
		Outdoor worker count	Annual number of workers engaged in agriculture, fishing, forestry, construction, and other outdoor‐related industries at the administrative dong level	Microdata Integrated Service
	Response	Accessibility to parks	Annual mean distance from the nearest facility to the centroid of the grid. Here, the centroid of the grid refers to the center point of each 100 m × 100 m grid cell provided by Korea's national gridded spatial framework (shorter distances imply better accessibility).	V‐WORLD
		Accessibility to medical facilities		LOCALDATA
		Accessibility to pharmacies		
		Accessibility to green spaces		Environmental Geographic Information Service
		Accessibility to water areas		
		Accessibility to heat shelters		Seoul Open Data Plaza
Dependent variable	Incidence rate of HRI		Monthly incidence rate of HRI per 10,000 people at the dong level	NHIS‐Big Data Platform

*Note.* Monthly data were extracted for the summer period (June–August).

### 4.3 Analysis Method

**Figure 2:**
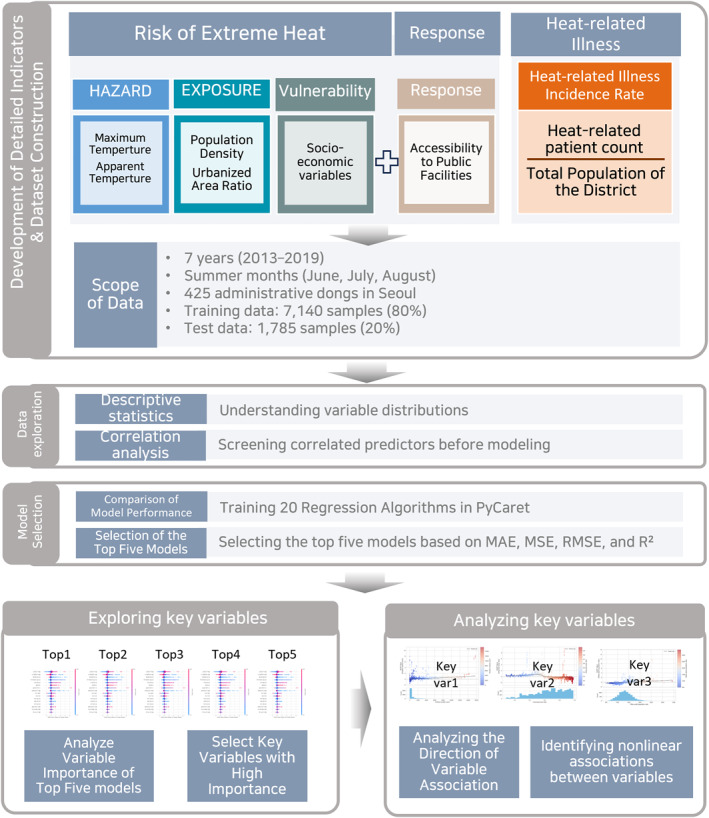
Research flow chart.

The analytical procedure of this study consisted of four steps, as illustrated in Figure 2. First, a data set was constructed based on the heatwave risk framework. Second, descriptive statistics and correlation analyses were performed to examine the distributions and relationships among the variables. Third, multiple machine‐learning algorithms were trained using the data set, and their performance metrics were compared. Finally, SHAP plots from the top five models were analyzed to identify the most influential variables and their directional effects on HRI incidence.

#### 4.3.1 Machine‐Learning Algorithms for Regression

We used PyCaret to develop and compare multiple regression models for predicting the HRI incidence rate. Regression algorithms were selected because the dependent variable is continuous, and evaluating multiple models is necessary to identify the approach with the best predictive performance. PyCaret, an open‐source automated machine‐learning library in Python, was employed to efficiently train, tune, and compare diverse high‐performance regression algorithms in a systematic and reproducible manner. In the first phase of the analysis, 20 regression algorithms were evaluated. Ten were linear models—Linear Regression, Ridge Regression, Lasso Regression, Elastic Net, Bayesian Ridge, Least Angle Regression, Lasso Least Angle Regression, Orthogonal Matching Pursuit, Huber Regressor, and Passive Aggressive Regressor—which are commonly applied under assumptions such as linearity, independence, homoscedasticity, and low multicollinearity (James et al., 2013). The remaining nonlinear models consisted of five boosting‐based algorithms (CatBoost Regressor, Light Gradient Boosting Machine Regressor, Extreme Gradient Boosting Regressor, Gradient Boosting Regressor, and AdaBoost Regressor), three tree‐based algorithms (Extra Trees Regressor, Random Forest Regressor, and Decision Tree Regressor), and one distance‐based algorithm (K‐Nearest Neighbors Regressor). A Dummy Regressor was also included as a baseline. While nonlinear models offer greater flexibility with fewer distributional assumptions, they may be more susceptible to overfitting without appropriate regularization (Labib, 2024; Wiemken & Kelley, 2020).

All models were first trained with PyCaret (Version 3.3.2) using default hyperparameters, followed by automated hyperparameter tuning using the tune_model() function. This function explores a predefined hyperparameter search space and identifies optimal configurations by evaluating candidate models through K‐fold cross‐validation. For each algorithm, the configuration demonstrating superior validation performance was selected as the final model. To improve predictive accuracy, *R* ^2^ was specified as the optimization metric. The hyperparameters applied in the finalized models are summarized in Table S1 of the Supporting Information S1. Model validation followed PyCaret's default 10‐fold cross‐validation protocol. The data set was randomly partitioned into 10 equal subsets; each subset was used once as the validation fold while the remaining nine were used for training. After completing all folds, the final model performance was calculated as the average of evaluation metrics across folds.

#### 4.3.2 SHAP as an Explainable AI Technique

We applied SHAP to interpret the results of the machine‐learning models and to quantify the contribution of individual predictors to HRI incidence. SHAP extends Shapley values from cooperative game theory to machine‐learning models, enabling consistent and interpretable attribution of feature effects on model predictions (Lundberg et al., 2018). Shapley values estimate a feature's contribution by comparing the model prediction with and without the feature across all possible feature combinations. The SHAP framework supports optimized algorithms such as Tree SHAP and Kernel SHAP, enabling efficient interpretation of complex machine‐learning models.

To facilitate interpretation, SHAP values were visualized using summary and dependence plots. Summary plots provide a global overview of feature importance by displaying SHAP values for all observations. In this study, dot‐type summary plots were primarily used, as they convey both the magnitude and direction of feature effects. Features are ranked by importance on the *y*‐axis, while SHAP values are shown on the *x*‐axis, with point colors representing the relative magnitude of feature values. This visualization enables simultaneous assessment of feature importance, effect direction, and value distribution.

Dependence plots were used to further examine the relationships between key predictors and HRI incidence. In these plots, the *x*‐axis represents the observed feature values and the *y*‐axis represents the corresponding SHAP values, allowing identification of potential non‐linear and threshold effects. These plots were used to examine how changes in individual variables were associated with increases or decreases in the predicted HRI incidence rate.

## 5 Results

### 5.1 Descriptive Statistics and Correlation of All Variables

Table 2 presents the descriptive statistics of all variables used in the analysis. All variables were calculated based on 8,925 community‐level observations. Temperature‐related variables showed similar mean and median values, indicating relatively symmetric distributions across communities. In contrast, most demographic, socioeconomic, and response‐related variables, as well as the HRI incidence rate, exhibited higher mean values than medians, reflecting right‐skewed distributions.

**Table 2:** Descriptive Statistics of the Variables

Detailed indicators (community Characteristics; Unit)	N	Mean	Std	Min	Q1	Median	Q3	Max
Maximum temperature (°C)	8925	33.89	2.04	29.79	32.37	33.77	35.07	39.03
Apparent temperature (°C)	8925	35.56	2.23	31.31	33.57	35.87	36.97	40.96
Population density (persons/10,000 m^2^)	8925	238.04	119.05	2.97	145.06	241.44	319.91	585.76
Urbanized area ratio (%)	8925	0.66	0.21	0.08	0.51	0.69	0.83	0.98
Older‐adult population ratio (per 10,000 population)	8925	1342.91	312.61	494.09	1131.23	1326.03	1528.15	3431.73
Infant and toddler population ratio (per 10,000 population)	8925	362.25	111.94	60.42	292.03	353.94	416.95	1491.04
Low‐income group ratio (per 10,000 population)	8925	255.68	216.92	0	126.14	215.42	322.98	1920.89
Older‐adult living alone ratio (per 10,000 population)	8925	320.87	157.07	0	221.39	297.44	387.87	1925.39
Disabled population ratio (per 10,000 population)	8925	402.41	145.71	32.95	320.49	398.76	463.3	1606.05
Outdoor worker count (persons)	8925	775.61	1623.92	0	71	201	636	15,720
Accessibility to parks (m)	8925	146.04	119.42	6.72	87.24	119.32	168.47	1478.41
Accessibility to medical facilities (m)	8925	460.31	272.52	145.26	278.8	377.89	553.62	2383.44
Accessibility to pharmacies (m)	8925	285.14	213.34	87.52	157.35	217.73	323.94	1613.55
Accessibility to green spaces (m)	8925	36.33	25.41	2.23	17.05	30.92	50.25	160.58
Accessibility to water areas (m)	8925	638.09	383.05	53.17	336.18	529.62	855.67	2057.39
Accessibility to heat shelters (m)	8925	303.18	201.53	97.18	188.66	243.7	339.25	1885.41
HRI incidence rate (cases/10,000 population)	8925	0.92	2.41	0	0	0.38	0.92	40.09

**Figure 3:**
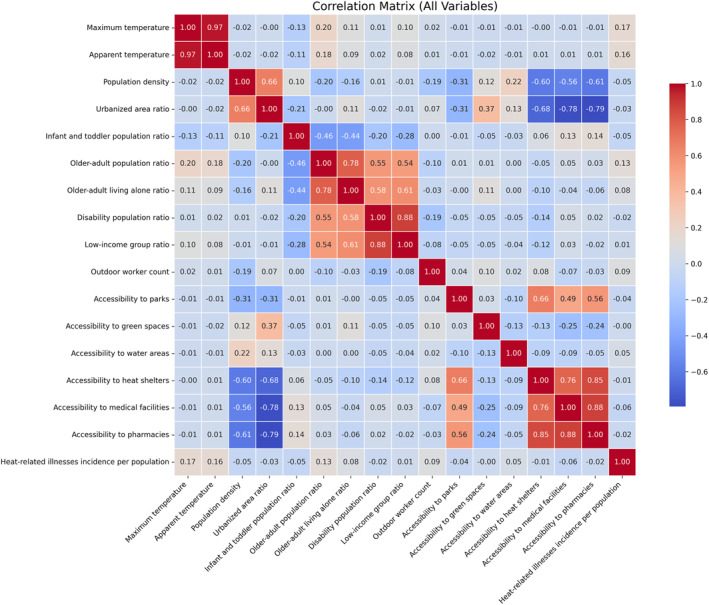
Correlation heatmap of all variables.

Pearson correlation analysis revealed that several variables exhibited strong linear associations (Figure 3). Among meteorological indicators, maximum temperature and apparent temperature showed a very high correlation (*r* ≈ 0.97), indicating that both variables capture almost identical thermal environmental characteristics. Within demographic and socioeconomic vulnerability indicators, high correlations were also observed between older‐adult population ratio and older adults living alone ratio (*r* ≈ 0.78), disability population ratio and low‐income group ratio (*r* ≈ 0.61), and population density and urbanized area ratio (*r* ≈ 0.66). These results suggest that certain vulnerability factors tend to coexist within the same communities. For response indicators, particularly strong correlations were found among accessibility‐related variables. Accessibility to medical facilities and accessibility to pharmacies exhibited a very high correlation (*r* ≈ 0.88), as did accessibility to heat shelters and accessibility to pharmacies (*r* ≈ 0.88). In addition, accessibility to water areas showed high correlations with accessibility to medical facilities (*r* ≈ 0.76) and accessibility to pharmacies (*r* ≈ 0.85), while accessibility to heat shelters also correlated strongly with accessibility to medical facilities (*r* ≈ 0.76). These results imply that the spatial distributions of these public facilities are likely to be similar. In contrast, correlations between HRI incidence and all other variables were very low, remaining below 0.2. This finding indicates that linear effects alone are insufficient to explain HRI risk, underscoring the need to account for nonlinear relationships and interactions among variables. Accordingly, SHAP‐based explainable machine‐learning techniques were applied in this study.

### 5.2 Machine‐Learning Training Results and Top Five Algorithms

The results obtained by applying the 20 regression models are presented in Table 3. Based on the final performance metrics, the top five models were identified as CatBoost Regressor (*R* ^2^ = 0.750), Extreme Gradient Boosting Regressor (*R* ^2^ = 0.740), Extra Trees Regressor (*R* ^2^ = 0.736), Random Forest Regressor (*R* ^2^ = 0.717), and Light Gradient Boosting Machine Regressor (*R* ^2^ = 0.717). All of these models are nonlinear tree‐based or boosting‐based algorithms, and they achieved *R* ^2^ values greater than 0.70, demonstrating high predictive accuracy. In addition, their RMSE values ranged from 1.02 to 1.11, which indicates strong predictive stability and relatively low prediction error.

**Table 3:** Comparison of Performance Among Regression Algorithms

Rank	Model	Linear/Nonlinear	Default/Tuned	MAE	MSE	RMSE	*R* ^2^
1	CatBoost Regressor	NL*	Default	0.56	1.039	1.019	0.75
2	Extreme Gradient Boosting Regressor	NL	Tuned	0.567	1.08	1.039	0.74
3	Extra Trees Regressor	NL	Default	0.578	1.095	1.047	0.737
4	Random Forest Regressor	NL	Default	0.589	1.177	1.085	0.717
5	Light Gradient Boosting Machine Regressor	NL	Tuned	0.589	1.178	1.086	0.717
6	Gradient Boosting Regressor	NL	Tuned	0.599	1.222	1.106	0.706
7	Decision Tree Regressor	NL	Tuned	0.68	1.63	1.277	0.608
8	K Neighbors Regressor	NL	Tuned	0.681	2.135	1.461	0.486
9	Bayesian Ridge Regression	L**	Tuned	0.945	3.879	1.97	0.067
10	Lasso Least Angle Regression	L	Tuned	0.939	3.879	1.97	0.067
11	Orthogonal Matching Pursuit	L	Tuned	0.951	3.881	1.97	0.066
12	Ridge Regression	L	Tuned	0.952	3.881	1.97	0.066
13	Linear Regression	L	Tuned	0.953	3.882	1.97	0.066
14	Least Angle Regression	L	Tuned	0.953	3.882	1.97	0.066
15	AdaBoost Regressor	NL	Tuned	0.876	3.993	1.998	0.039
16	Huber Regressor	L	Tuned	0.774	4.097	2.024	0.014
17	Elastic Net	L	Tuned	0.984	4.163	2.04	−0.002
18	Lasso Regression	L	Tuned	0.984	4.163	2.04	−0.002
19	Dummy Regressor	baseline	Default	0.984	4.163	2.04	−0.002
20	Passive Aggressive Regressor	L	Tuned	1.124	4.503	2.122	−0.084

*Note.* NL* = nonlinear; L** = linear; MAE = mean absolute error; MSE = mean squared error; RMSE = root mean square error.

In contrast, the performance of linear models was substantially lower. Most linear algorithms showed *R* ^2^ values near 0.066 and RMSE values between 1.96 and 2.04, indicating limited predictive capability. This suggests that HRI incidence in this study is likely governed by nonlinear relationships that cannot be sufficiently captured through linear assumptions alone.

In summary, nonlinear tree‐based models and boosting‐based models significantly outperformed linear models. Therefore, the top five models were selected for subsequent analyses, including the interpretation of feature contributions and the directional effects of influential variables.

### 5.3 Variable Importance Among the Top Five Models

The results of the variable importance analysis across the top‐performing five models are presented in Table 4. Urbanized area ratio and older‐adult population ratio were included in the top five features in all models. Apparent temperature, accessibility to medical facilities, outdoor worker count, and accessibility to water areas were included in the top five features in at least three models. The variable rankings were further examined based on the IPCC (2023) Hazard–Exposure–Vulnerability–Response framework. Among the hazard indicators, apparent temperature showed higher rankings than maximum temperature. For the exposure indicators, urbanized area ratio was ranked higher than population density. For the vulnerability indicators, older‐adult population ratio and outdoor worker count ranked higher than the other vulnerability‐related variables. For the response indicators, accessibility to medical facilities and accessibility to water areas were the highest‐ranking features. In contrast, low‐income group ratio, older‐adult living alone ratio, infant and toddler population ratio, accessibility to parks, and accessibility to green spaces consistently exhibited low rankings across all models.

**Table 4:** Variable Importance Rankings Across the Top Five Models

Component	Variable	CatBoost regressor	Extreme gradient boosting regressor	Extra trees regressor	Random forest regressor	Light gradient boosting machine regressor
**E**	Urbanized area ratio	**1**	**1**	**2**	**4**	**1**
**V**	Older‐adult population ratio	**3**	**2**	**3**	**3**	**2**
**R**	Accessibility to medical facilities	**2**	**3**	**5**	6	**3**
**R**	Accessibility to water areas	**4**	7	**1**	**2**	7
**H**	Apparent temperature	11	**4**	**4**	**1**	8
**V**	Outdoor worker count	**5**	9	7	**5**	**4**
H	Maximum temperature	7	5	6	7	5
R	Accessibility to heat shelters	6	8	8	8	6
V	Disability population ratio	9	6	11	9	9
R	Accessibility to pharmacies	8	10	12	12	10
E	Population density	10	16	9	10	14
V	Infant and toddler population ratio	14	11	14	11	12
R	Accessibility to parks	13	12	13	13	11
R	Accessibility to green spaces	12	14	10	15	13
V	Low‐income group ratio	15	13	16	14	16
V	Older‐adult living alone ratio	16	15	15	16	15

*Note.* The Component column indicates H = hazard, E = exposure, V = vulnerability, and R = response. Bold values indicate variables ranked within the top five in importance.

### 5.4 Detailed Analysis of Variable Relationships

**Figure 4:**
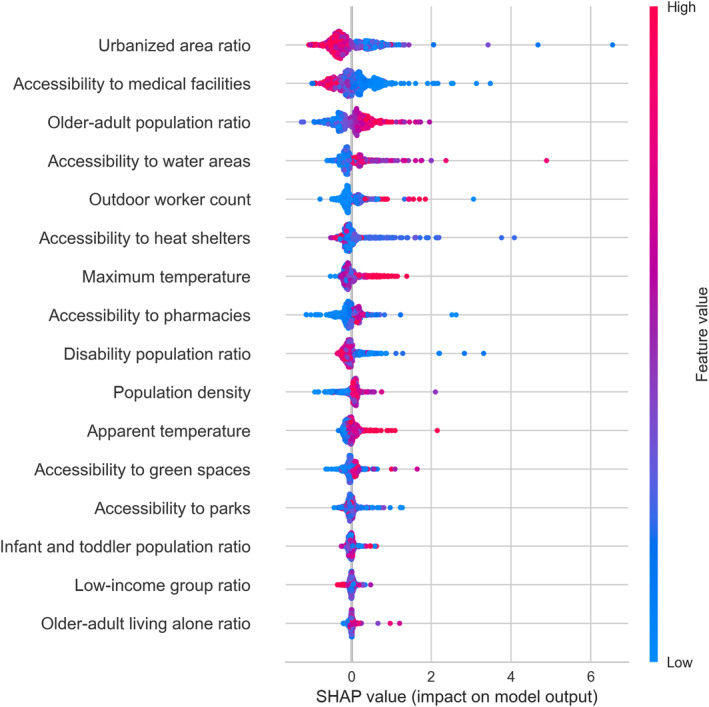
Summary dot plot of CatBoost Regressor.

To examine how the six key variables identified in the previous section were associated with HRI incidence, we first generated SHAP summary dot plots. Since the direction and magnitude of effects were highly consistent across the five machine‐learning models, we selected the CatBoost Regressor—the model with the best predictive performance—as a representative example. Figure 4 presents the SHAP summary dot plot, which visualizes the directionality of these variables. The SHAP summary dot plots for the remaining four models are provided in Figures S1–S5 of the Supporting Information S1.

In Figure 4, higher urbanized area ratio and better accessibility to medical facilities (i.e., shorter distance) were associated with higher HRI incidence. Higher apparent temperatures, greater distance from water areas, and a higher older‐adult population ratio were also associated with increased incidence. For outdoor worker count, however, the direction of the effect was not clearly distinguishable from the summary plot alone.

**Figure 5:**
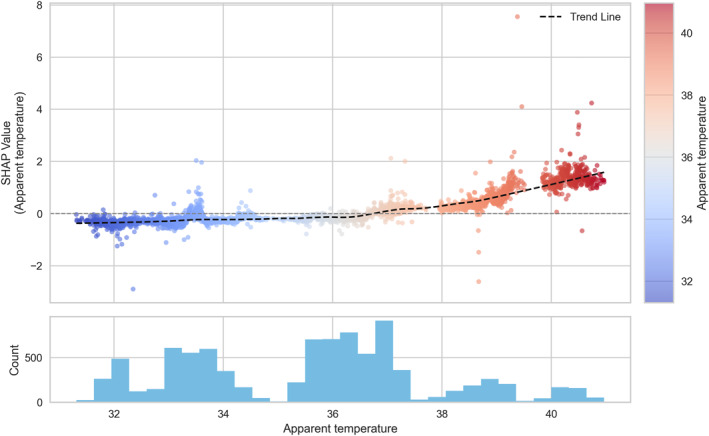
Dependence plot of apparent temperature.

**Figure 6:**
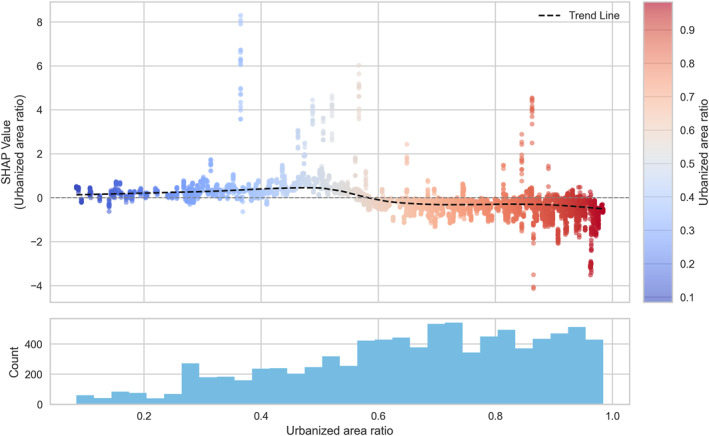
Dependence plot of urbanized area ratio.

**Figure 7:**
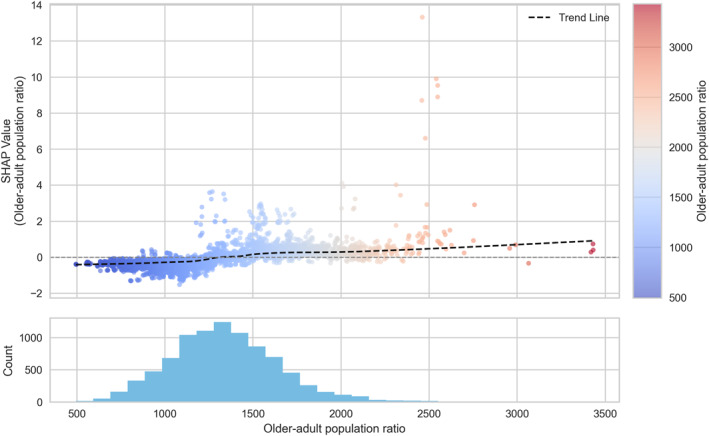
Dependence plot of older‐adult population ratio.

**Figure 8:**
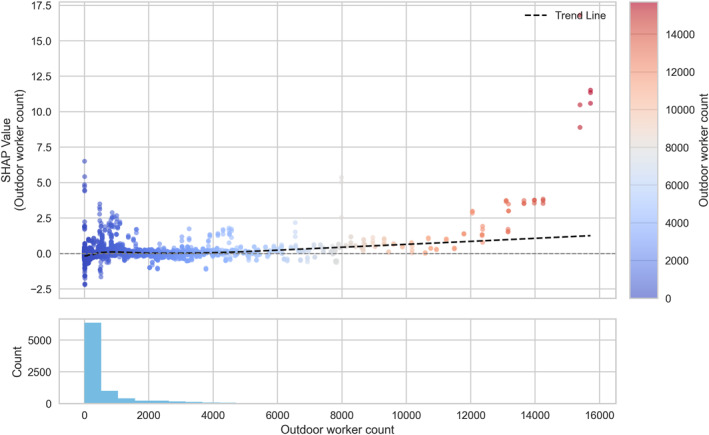
Dependence plot of outdoor worker count.

**Figure 9:**
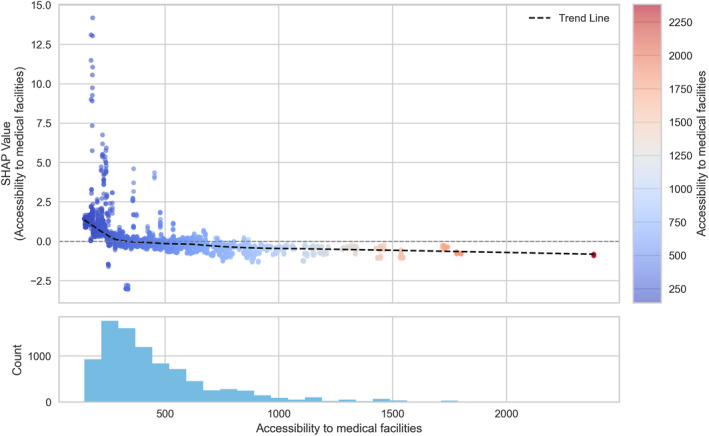
Dependence plot of accessibility to medical facilities.

**Figure 10:**
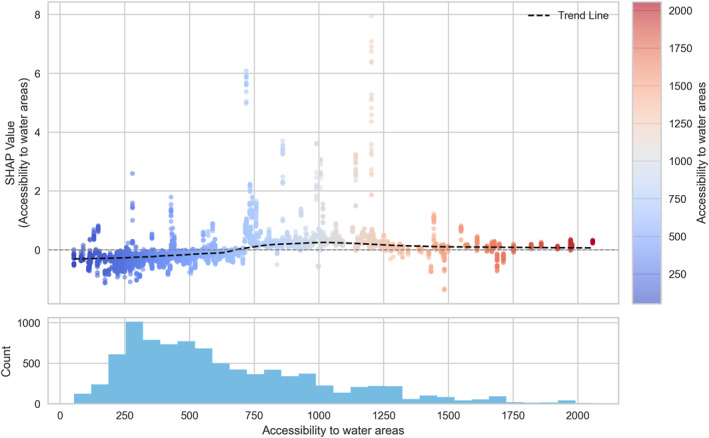
Dependence plot of accessibility to water areas.

To explore these nonlinear relationships in greater detail, SHAP dependence plots were generated for the six most influential variables (Figures 5, 6, 7, 8, 9, 10), accompanied by histograms to illustrate the distribution of each predictor.

Among the hazard indicators, apparent temperature (Figure 5) showed a clear threshold‐type relationship with HRI risk, as reflected in SHAP values. SHAP values remained low below approximately 36°C, but sharply increased above this threshold, indicating a strong escalation of HRI risk under extreme thermal stress conditions. For the exposure indicators, the urbanized area ratio (Figure 6) exhibited a nonlinear pattern with a change in effect direction. SHAP values were slightly positive when the ratio was below 0.6, but gradually shifted negative beyond 0.6, indicating a higher risk in less urbanized neighborhoods. For the vulnerability indicators, the older‐adult population ratio (Figure 7) showed a weak but consistently positive relationship with HRI incidence. SHAP values remained near zero below approximately 1,500 persons per 100,000 population, but increased steadily thereafter, with a notable high‐risk cluster emerging above 2,500 persons per 100,000. Likewise, the number of outdoor workers (Figure 8) contributed increasingly to risk as values rose, with a substantial high‐risk group appearing in districts with more than 10,000 outdoor workers. Among the response‐related indicators, accessibility to medical facilities (Figure 9)—defined as distance to the nearest hospital—suggested a slight tendency toward higher risk in areas where medical services were more easily accessible. This pattern may reflect better detection and reporting in areas with close medical access. Finally, accessibility to water areas (Figure 10) demonstrated a distance‐based effect: HRI risk increased as distance grew within roughly 1 km, indicating reduced availability of cooling environments in distant neighborhoods. Beyond this range, the contribution to risk approached zero.

## 6 Discussion

This study was conducted to provide scientific evidence for proactive responses to HRI risks caused by climate change by simultaneously considering regional physical environments and sociodemographic conditions and applying explainable artificial intelligence techniques. As a result, the variables most strongly associated with the incidence of HRI were apparent temperature, urbanized area ratio, older‐adult population ratio, outdoor worker count, and accessibility to water areas. In other words, the findings highlight that multidimensional factors—hazard (apparent temperature), exposure (urbanization), vulnerability (older adults and outdoor workers), and response (accessibility to water and medical facilities)—work together to determine HRI risk.

Several machine‐learning–based heat–health studies have identified environmental and socioeconomic factors—particularly heat exposure indicators (e.g., temperature and humidity), urban development intensity, and the proportion of older adults—as major contributors to heat‐related health risks. In line with these findings, our results also demonstrated the strong influence of these factors, while additionally highlighting the role of outdoor workers and proximity to water areas, which have been relatively underrepresented in previous data‐driven analyses (Foroutan et al., 2025; Guo et al., 2025; Yang et al., 2025).

Among the hazard indicators, apparent temperature demonstrated stronger relevance in model predictions of HRI incidence compared to maximum temperature. This may be because apparent temperature incorporates humidity and thus may better represent the thermal environment actually experienced by humans. A clear threshold was identified around 36°C, above which the risk appears to increase rapidly. This pattern is consistent with existing biomedical evidence suggesting that health impacts tend to accelerate when heat stress exceeds the physiological limits of the human body (Cramer et al., 2022; Filingeri & Koch Esteves, 2025). Among the exposure indicators, the urbanized area ratio was found to be a more important variable than population density. The model suggested that communities with lower urbanized area ratios tended to be associated with higher predicted HRI incidence, a result that contrasts with some previous studies (Lee et al., 2019, 2022). Higher incidence rates in less‐urbanized areas observed in our study may be due to more frequent outdoor activities and potentially lower accessibility to cooling infrastructure or protective facilities.

Regarding vulnerability indicators, the proportion of the older‐adult population and the number of outdoor workers were identified as important variables, consistent with previous research (Åström et al., 2011; Bunker et al., 2016; Hondula et al., 2015). These findings may be explained by distinct vulnerability mechanisms. Older adults are biologically more susceptible to heat stress due to reduced thermoregulatory capacity, a higher prevalence of chronic conditions, and limited physiological adaptability. Outdoor workers, on the other hand, often need to remain active outdoors during periods of extreme heat because of job‐related demands, which can lead to sustained exposure to high‐temperature environments and an increased risk of HRI. Other vulnerability variables, including the proportions of infants, low‐income households, and older adults living alone, showed low importance and did not demonstrate clear directional patterns. This does not imply that these groups are less vulnerable; rather, their heat‐related risks may be mitigated by contextual factors such as caregiver support, access to air‐conditioned indoor environments, or other community‐level protective resources.

Among the response indicators, accessibility to medical facilities and water areas were identified as the most important variables, while the importance of accessibility to pharmacies, parks, and green spaces was low. Higher HRI incidence observed in areas with better medical accessibility may reflect detection bias, as mild cases are more likely to be diagnosed and reported compared to regions with poor healthcare access (Lee et al., 2016). Accessibility to water areas exhibited a distance‐dependent, nonlinear association with HRI incidence. Within approximately 1 km, increasing distance was associated with higher HRI incidence, consistent with potential cooling and microclimate benefits provided by nearby water bodies (Hao et al., 2016; Park et al., 2011). Beyond this range, the association weakened, implying that such protective effects are spatially limited to walkable activity areas. The limited importance of accessibility to pharmacies, green spaces, and parks can be interpreted as potentially reflecting a tendency for people to prefer indoor, air‐conditioned environments during heatwaves, thereby reducing direct exposure (Bowler et al., 2010; Zhou et al., 2014).

From a policy perspective, the findings of this study suggest that heatwave response strategies should be differentiated according to the characteristics of key explanatory variables. First, apparent temperature showed the strongest association with HRI risk and exhibited a threshold‐type increase above a critical level. This suggests that short‐term heatwave responses may benefit from using apparent temperature as a primary reference indicator, with strengthened heat‐alert issuance and response mechanisms during periods of extreme thermal stress. Furthermore, the higher HRI risk observed in areas with a lower urbanized area ratio suggests that these regions may be relatively vulnerable in terms of access to heat‐response services and infrastructure. This implies that peri‐urban or less‐urbanized areas could warrant additional policy attention with respect to the allocation and accessibility of heat‐response resources. At the same time, the older‐adult population ratio and the number of outdoor workers were consistently positively associated with HRI risk, suggesting that these groups represent populations requiring more focused consideration in heatwave response planning. Areas with higher concentrations of older adults and outdoor workers may therefore warrant prioritized attention, given their biological susceptibility and occupational exposure, respectively. Finally, accessibility to water areas exhibited a distance‐limited mitigating effect, with reductions in HRI risk observed only within approximately 1 km. This suggests that the effectiveness of water areas as heat‐mitigation resources may depend on practical, walkable accessibility rather than mere presence, highlighting the importance of enhancing connectivity to water areas within daily activity ranges as part of long‐term heat‐adaptation strategies.

This study addresses limitations of previous heat‐health research that primarily focused on individual‐level or single environmental determinants by integratively evaluating community‐level physical environments, social vulnerability, and response capacity within the IPCC AR6 Hazard–Exposure–Vulnerability–Response framework. By applying explainable artificial intelligence techniques capable of capturing nonlinear dynamics and feature interactions, this study delivered results with both high predictive performance and strong interpretability, thereby enhancing their policy relevance. The findings of this study provide practical evidence that can inform spatially targeted intervention strategies. In addition, this study contributes new empirical evidence on the threshold effect of apparent temperature (approximately 36°C), the locally constrained cooling effects of water areas (within an approximate 1 km radius), and the relationship between healthcare accessibility and potential detection bias, all of which offer important insights for the design of effective heat–health response strategies.

Several limitations should be considered when interpreting the results of this study. First, meteorological data were spatially interpolated from observation stations using kriging. Although this approach is widely applied, it may not fully capture localized microclimatic variability, particularly in heterogeneous urban environments. Second, because the analysis focused exclusively on Seoul, the generalizability of the findings to smaller cities or rural areas, where urban structures and population characteristics differ substantially, may be limited. Third, the response component was operationalized primarily through physical infrastructure accessibility, and thus did not account for existing social support mechanisms, such as cooling‐cost assistance or targeted outreach programs, which may also influence community resilience. Finally, HRI data were derived from medical utilization and reporting records; therefore, potential detection bias related to healthcare accessibility or care‐seeking behavior may have led to an underestimation of risks in underserved areas. Future research should seek to expand the analytical scope to diverse regional contexts, incorporate additional behavioral and social variables, and utilize higher‐resolution environmental data to further improve understanding of the mechanisms underlying community‐level heat‐related health risks.

## 7 Conclusion

This study highlights the critical role of community‐level characteristics—particularly thermal environments, exposure conditions, and sociodemographic vulnerabilities—in shaping HRI risks. It identifies key drivers such as apparent temperature, urbanized area ratio, older‐adult population ratio, outdoor worker count, and accessibility to water areas, and provides actionable evidence for targeted heatwave adaptation strategies. Future efforts should focus on strengthening cooling infrastructure, improving accessibility to protective facilities, and prioritizing support for heat‐vulnerable populations as climate change continues to intensify heat risks.

## Conflict of Interest

The authors declare no conflicts of interest relevant to this study.

## Supporting information

Supporting Information S1

## Data Availability

The data sets used in this study are publicly available through various Korean government agencies; however, most of the data portals do not provide English‐language interfaces. Maximum temperature and apparent temperature data were obtained from the Korea Meteorological Administration Data Portal (https://data.kma.go.kr/data/grnd/selectAwsRltmList.do?pgmNo=56). Users can select the desired time unit from the “자료형태” (data type) dropdown menu, check the boxes for the target region and the “기온” (temperature) and “습도” (humidity) variables, and download the data. Apparent temperature was calculated based on the retrieved temperature and humidity data. Population‐related data, including population density, older‐adult population ratio, and infant and toddler population ratio, were accessed through Statistics Korea (https://kosis.kr/statHtml/statHtml.do?sso=ok&returnurl=https%3A%2F%2Fkosis.kr%3A443%2FstatHtml%2FstatHtml.do%3Flist_id%3DA_7%26obj_var_id%3D%26seqNo%3D%26tblId%3DDT_1B04005N%26vw_cd%3DMT_ZTITLE%26orgId%3D101%26path%3D%252FstatisticsList%252FstatisticsListIndex.do%26conn_path%3DMT_ZTITLE%26itm_id%3D%26lang_mode%3Dko%26scrId%3D%26). Users can click the “조회설정” (Query Settings) button, select the desired year and region, and then click “다운로드” (Download) to export the data. Sociodemographic data, such as the low‐income group ratio, were obtained from:
https://data.seoul.go.kr/dataList/DT201004O1100342013/S/2/datasetView.do
https://data.seoul.go.kr/dataList/DT201004O1100342015/S/2/datasetView.do Data on the disabled population ratio, older adults living alone, and heat shelter locations were obtained from:
https://data.seoul.go.kr/dataList/10577/S/2/datasetView.do
https://data.seoul.go.kr/dataList/10176/S/2/datasetView.do
https://data.seoul.go.kr/dataList/OA‐21065/S/1/datasetView.do To access these data sets, users must click the “일괄설정” (Batch Settings) button, select the desired year and region, click “적용” (Apply), and then click “다운로드” (Download). Environmental spatial data, including urbanized area ratio, green spaces, and water areas, were obtained from the Environmental Geographic Information Service (https://egis.me.go.kr/req/intro.do). Users must register, log in, go to “자료신청” (Data Request) → “자료신청 내역” (Data Request Status), submit the application form, and download the data upon approval. Accessibility‐related data sets were not obtained as pre‐processed accessibility indicators. Instead, spatial data sets containing the coordinates of facilities were downloaded and used to calculate accessibility through spatial analysis. Specifically: Medical facilities and pharmacies: https://www.localdata.go.kr/devcenter/dataDown.do?menuNo=20001
Parks: https://www.vworld.kr/dtmk/dtmk_ntads_s002.do?svcCde=MK&dsId=30289
Heat shelters: https://data.seoul.go.kr/dataList/OA‐21065/S/1/datasetView.do
Green/water areas: https://egis.me.go.kr/req/intro.do Heat‐related illness data were obtained from the National Health Insurance Service (NHIS) Big Data Platform (https://nhiss.nhis.or.kr). After user registration, researchers must access the “연구과제 (Research Projects)” section and submit an application through the “맞춤형 연구 DB 신청 (Customized Research Database Application)” menu by following the designated procedures and obtaining approval prior to data access. As most of the above portals are only in Korean, non‐Korean speakers are advised to use browser‐based translation tools (e.g., Google Translate) and follow the data set titles and procedures described above. The processed data set used in this study, excluding the heat‐related illness incidence data, has been uploaded to Zenodo (Park & Kang, 2025; https://doi.org/10.5281/zenodo.17959038).
