# Supplementary material for: Do Community Characteristics Explain Heat‐Related Illness in Seoul, Korea?
Source: Geohealth. 2026 Mar 23;10(3):e2025GH001580. doi: 10.1029/2025GH001580 (PMC13093577; doi:10.1029/2025GH001580)
Supplement: Supplementary file 1 — Supporting Information S1 [file GH2-10-e2025GH001580-s001.docx]

*GeoHealth*

Supporting Information for

**Do Community Characteristics Explain Heat-Related Illness in Seoul, Korea?**

Minyeong Park, Jung Eun Kang

Department of Urban Planning and Engineering, Pusan National University

**Contents of this file**

Table S1

Figures S1 to S5

**Introduction**

This appendix provides additional technical details supporting the machine-learning analysis. It includes the hyperparameters used to train each finalized model (Table S1), extracted directly from the model objects (via get_params() in scikit-learn and equivalent interfaces for CatBoost, XGBoost, and LightGBM). Only parameters explicitly involved in model training are listed to ensure clarity and reproducibility. In addition, SHAP summary dot plots for the top-performing models (Figure S1~S5) are presented to illustrate the relative importance and directional effects of key predictors on heat-related illness incidence, with color gradients indicating feature magnitude and horizontal dispersion representing contribution strength.

**Table S1.** Final hyperparameter settings used in model training

| Model | Hyperparameters |
| --- | --- |
| AdaBoost Regressor (AdaBoostRegressor) | estimator='nan', learning_rate=1e-07, loss='exponential', n_estimators=280, random_state=42 |
| Bayesian Ridge Regression (BayesianRidge) | alpha_1=1e-06, alpha_2=1e-06, alpha_init='nan', compute_score=False, copy_X=True, fit_intercept=True, lambda_1=1e-06, lambda_2=1e-06, lambda_init='nan', n_iter='deprecated', normalize='deprecated', tol=0.001, verbose=False |
| CatBoost Regressor (CatBoostRegressor) | loss_function='RMSE', depth=6, l2_leaf_reg=6, iterations=254, learning_rate=0.05, random_seed=42, thread_count=-1, verbose=False, task_type='CPU', random_state=42 |
| Decision Tree Regressor (DecisionTreeRegressor) | ccp_alpha=0.0, criterion='mse', max_depth=5, max_features=1.0, max_leaf_nodes=None, min_impurity_decrease=0.0005, min_impurity_split=None, min_samples_leaf=6, min_samples_split=9, min_weight_fraction_leaf=0.0, presort='deprecated', random_state=42, splitter='best' |
| Dummy Regressor (DummyRegressor) | constant='nan', quantile='nan', strategy='mean' |
| Elastic Net (ElasticNet) | alpha=1.0, copy_X=True, fit_intercept=True, l1_ratio=0.5, max_iter=1000, normalize='deprecated', positive=False, precompute=False, random_state=42, selection='cyclic', tol=0.0001, warm_start=False |
| Extra Trees Regressor (ExtraTreesRegressor) | bootstrap=False, ccp_alpha=0.0, criterion='squared_error', max_depth=None, max_features=1.0, max_leaf_nodes=None, min_impurity_decrease=0.0, min_samples_leaf=1, min_samples_split=2, min_weight_fraction_leaf=0.0, n_estimators=200, n_jobs=-1, oob_score=False, random_state=42, verbose=0, warm_start=False |
| Gradient Boosting Regressor (GradientBoostingRegressor) | alpha=0.9, ccp_alpha=0.0, criterion='friedman_mse', init='nan', learning_rate=0.05, loss='ls', max_depth=3, max_features=None, max_leaf_nodes=None, min_impurity_decrease=0.0, min_samples_leaf=1, min_samples_split=2, min_weight_fraction_leaf=0.0, n_estimators=200, n_iter_no_change=None, random_state=42, subsample=1.0, tol=0.0001, validation_fraction=0.1, verbose=0, warm_start=False |
| Huber Regressor (HuberRegressor) | alpha=0.1, epsilon=1.9, fit_intercept=True, max_iter=100, tol=1e-05, warm_start=False |
| K-Nearest Neighbors Regressor (KNeighborsRegressor) | algorithm='auto', leaf_size=30, metric='minkowski', metric_params='nan', n_jobs=-1, n_neighbors=8, p=2, weights='uniform' |
| Least Angle Regression (Lars) | copy_X=True, eps=2.220446049250313e-16, fit_intercept=True, max_iter='nan', n_nonzero_coefs=500, normalize='deprecated', positive=False, precompute='auto', random_state=42, verbose=False |
| Lasso Regression (Lasso) | alpha=1.0, copy_X=True, fit_intercept=True, max_iter=1000, normalize='deprecated', positive=False, precompute=False, random_state=42, selection='cyclic', tol=0.0001, warm_start=False |
| LightGBM Regressor (LGBMRegressor) | boosting_type='gbdt', class_weight='nan', colsample_bytree=1.0, n_estimators=100, num_leaves=31, min_child_samples=20, subsample=1.0, learning_rate=0.1, max_depth=-1, n_jobs=0, reg_alpha=0.0, reg_lambda=0.0, random_state=42, feature_fraction=0.8, bagging_freq=3, bagging_fraction=0.8 |
| Lasso Lars Regression (LassoLars) | alpha=0.01, copy_X=True, eps=5e-05, fit_intercept=True, fit_path=True, jitter=None, max_iter=500, n_nonzero_coefs=None, normalize='deprecated', positive=False, precompute='auto', random_state=42, verbose=False |
| Linear Regression (LinearRegression) | copy_X=True, fit_intercept=True, n_jobs=-1, positive=False |
| Orthogonal Matching Pursuit (OrthogonalMatchingPursuit) | fit_intercept=True, max_iter=None, n_nonzero_coefs=11, normalize='deprecated', precompute='auto', tol='nan' |
| Passive Aggressive Regressor (PassiveAggressiveRegressor) | C=1.0, average=False, early_stopping=False, epsilon=0.1, fit_intercept=True, loss='epsilon_insensitive', max_iter=1000, n_iter_no_change=5, random_state=42, shuffle=True, tol=0.001, validation_fraction=0.1, verbose=0, warm_start=False |
| Random Forest Regressor (RandomForestRegressor) | bootstrap=True, ccp_alpha=0.0, criterion='squared_error', max_depth=None, max_features=1.0, max_leaf_nodes=None, min_impurity_decrease=0.0, min_samples_leaf=1, min_samples_split=2, min_weight_fraction_leaf=0.0, n_estimators=300, n_jobs=-1, oob_score=False, random_state=42, verbose=0, warm_start=False |
| Ridge Regression (Ridge) | alpha=8.63, copy_X=True, fit_intercept=True, max_iter=None, normalize='deprecated', positive=False, random_state=42, solver='auto', tol=0.0001 |
| Extreme Gradient Boosting Regressor (XGBRegressor) | objective='reg:squarederror', base_score=0.5, booster='gbtree', colsample_bylevel=1, colsample_bytree=1, gamma=0, learning_rate=0.05, max_depth=6, min_child_weight=1, n_estimators=300, reg_alpha=0, reg_lambda=1, subsample=0.8, random_state=42, tree_method='auto', verbosity=0 |

**
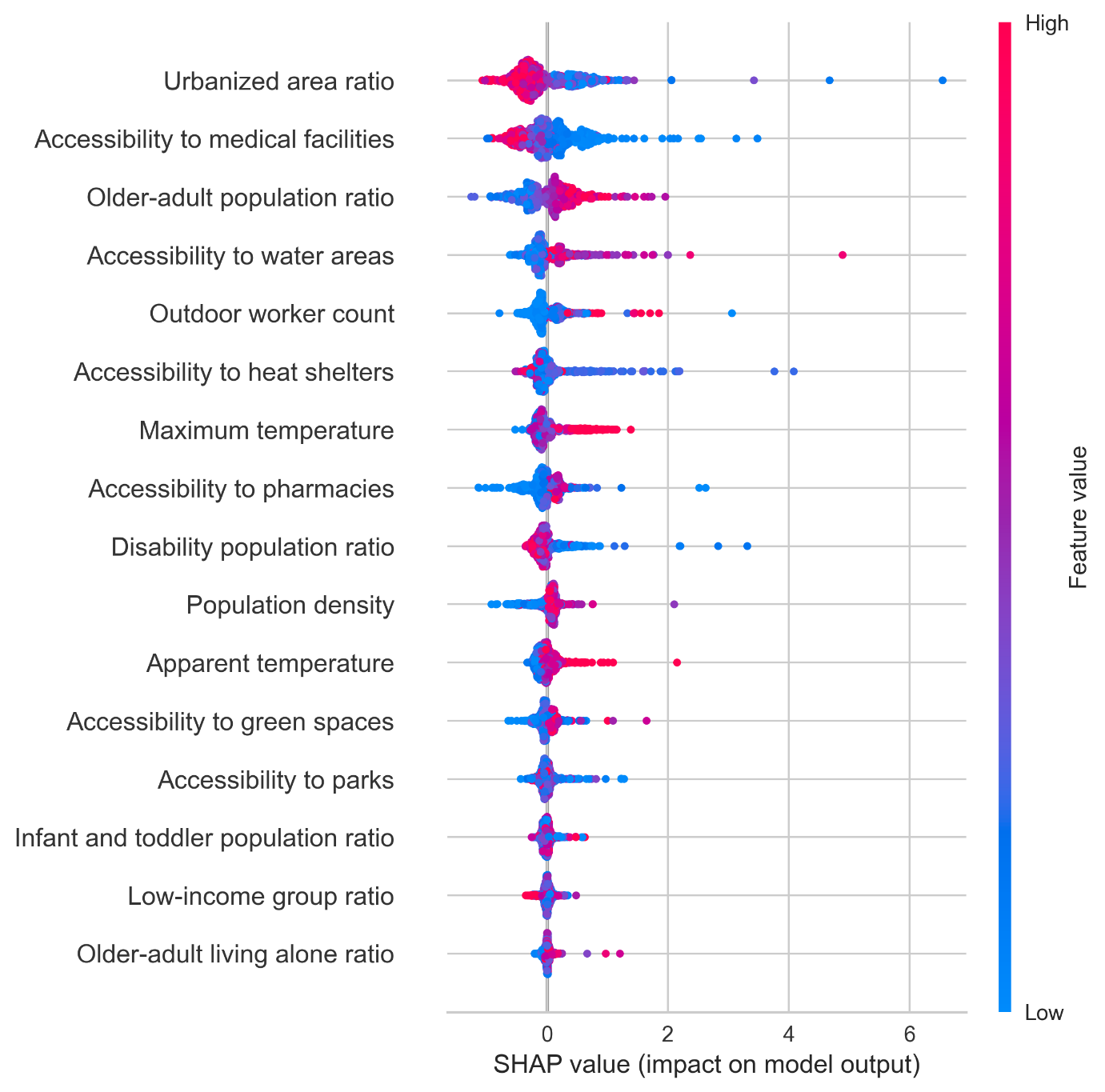
**

**Figure S1.** SHAP summary dot plot for CatBoost Regressor


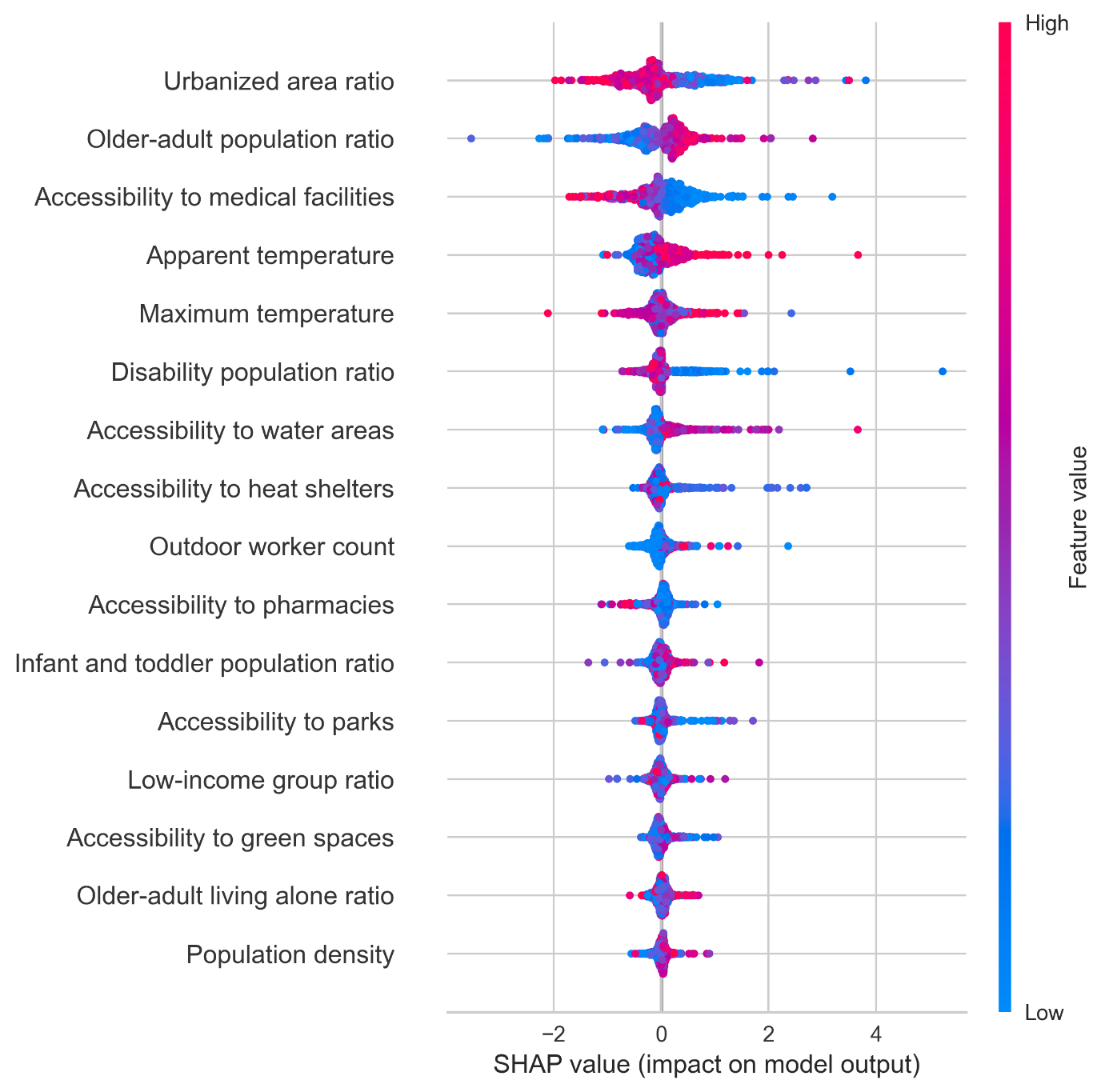


Figure S2. SHAP summary dot plot for Extreme Gradient Boosting Regressor


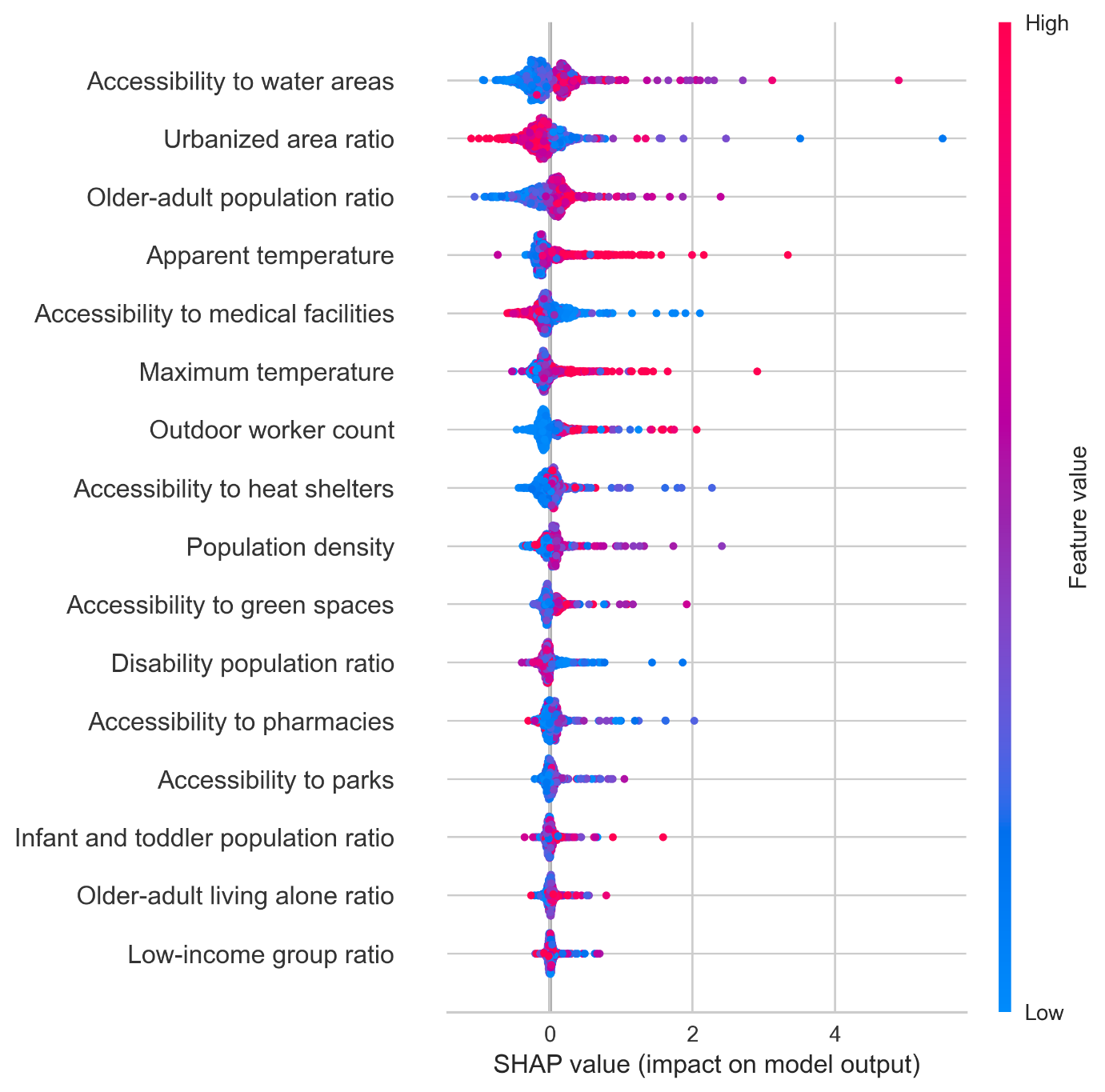


**Figure S3.** SHAP summary dot plot for Extra Trees Regressor


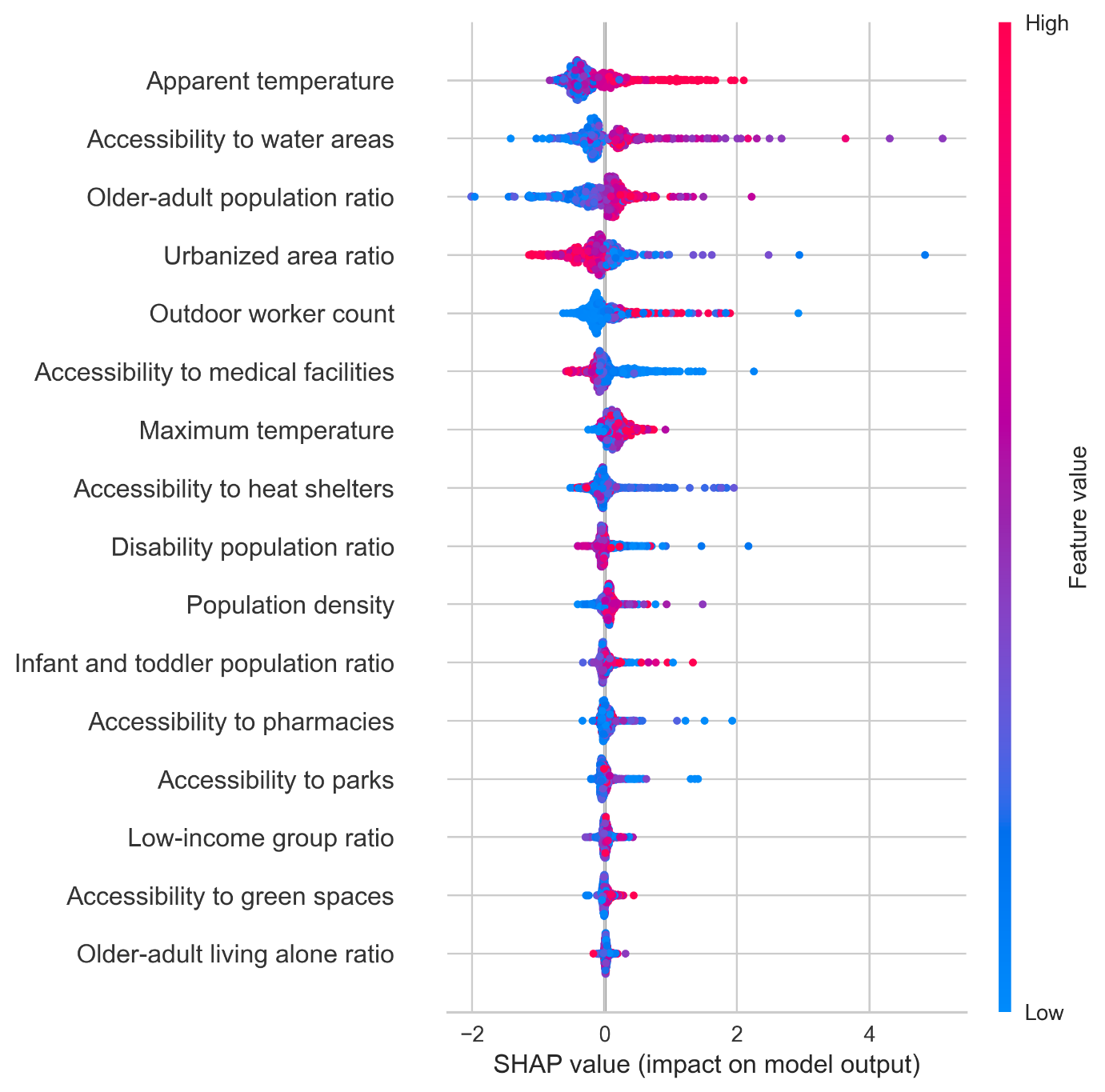


**Figure S4.** SHAP summary dot plot for Random Forest Regressor


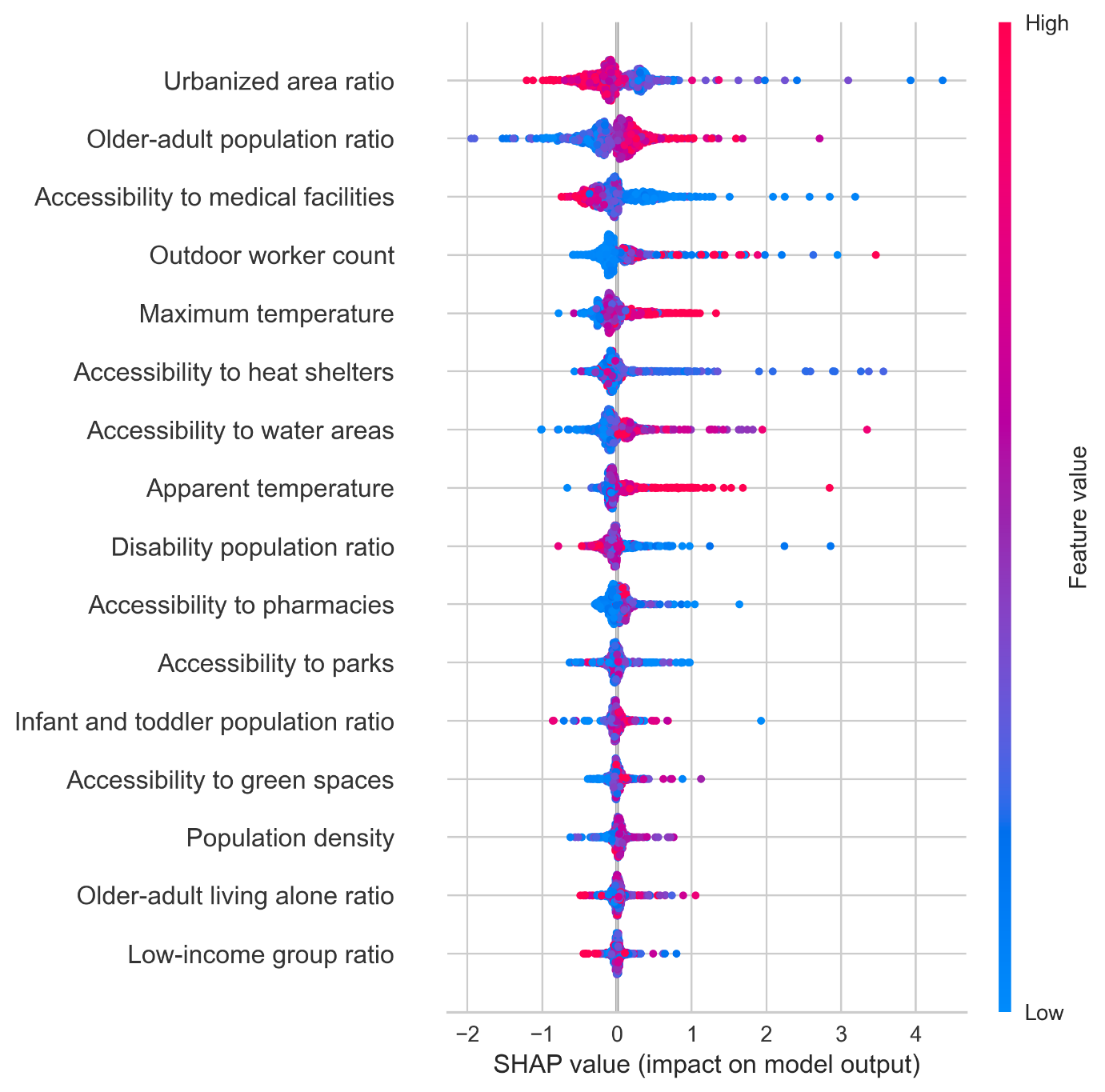


**Figure S5.** SHAP summary dot plot for Light Gradient Boosting Machine Regressor
